# Core–Shell Microgels at Air/Water Interfaces: Role of Interfacial Tension in Monolayer Evolution

**DOI:** 10.1021/acs.langmuir.4c05050

**Published:** 2025-03-31

**Authors:** Yichu Zhou, Jérôme J. Crassous, Matthias Karg

**Affiliations:** † Institut für Physikalische Chemie I: Kolloide und Nanooptik, Heinrich-Heine-Universität Düsseldorf, Universitätsstr. 1, 40225 Düsseldorf, Germany; ‡ Institut für Physikalische Chemie, RWTH Aachen University, Landoltweg 2, 52074 Aachen, Germany; § Physical Chemistry of Functional Polymers, Martin Luther University Halle-Wittenberg, Institute of Chemistry, 06120 Halle (Saale), Germany

## Abstract

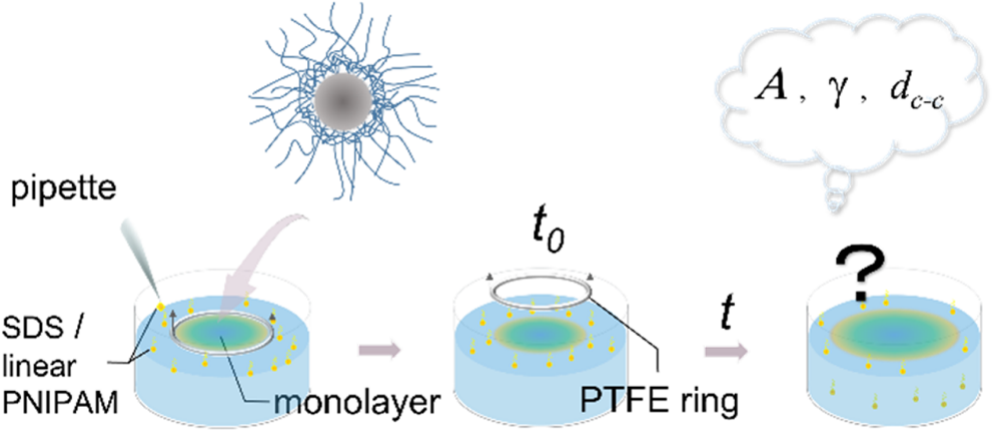

Core–shell microgels with rigid cores and soft, deformable hydrogel shells can assemble at air–water interfaces, forming freely floating monolayers. The strong adsorption at such interfaces is related to the reduction in interfacial tension, which also causes the microgels to deform laterally. The degree of this deformation is typically controlled through applied surface pressure. Until now, surprisingly little has been known about the impact of interfacial tension imbalances between interfacial areas covered with a microgel monolayer and microgel-free areas in the surroundings. In this work, we systematically study the monolayer evolution at air/water interfaces in dependence of interfacial tension controlled by the addition of sodium dodecyl sulfate or linear poly-*N*-isopropylacrylamide homopolymer to the free area. We do this by globally monitoring the evolution of the area of freely floating monolayers. Macroscopic changes are also related to the local microstructure studied by atomic force microscopy (AFM). Depending on the interfacial tension imbalance, the monolayer either expands, shrinks, or maintains its conformation. The kinetics of monolayer expansion is compared for core–shell microgels with the same silica core and varying cross-linker densities. Our study reveals the impact of interfacial tension on the behavior of microgel monolayers at liquid interfaces and also provides useful insights into controlling the two-dimensional (2D) microstructure without the need for a Langmuir trough.

## 1 Introduction

The (self-)assembly of nanoparticle (NP) building blocks into structurally defined superstructures is an essential step toward the development of new functional materials.^1^ Despite the influence of building block size, shape, and their dispersities, the surface chemistry crucially determines the assembly behavior and structure. Examples comprise electrostatic assembly between oppositely charged surfaces, either between individual NPs^2−5^ or NPs and substrates,^6−8^ DNA-guided assembly,^9,10^ and crystallization-induced assembly of polymer ligand-stabilized NPs.^11^ Synthetic and natural polymers are an important class of ligands where not only interactions between NPs can be tailored but also control over interparticle distances is possible simply via the molar mass of the polymers.^12−15^ Depending on the grafting density and ligand length, the resulting building blocks become soft and deformable. In recent years, NPs decorated with such soft shells have received increasing interest for their intriguing assembly behavior at solid and fluid interfaces.^16^ Fluid interface-assisted (self-)assembly is particularly appealing for achieving two-dimensional (2D) superstructures of NPs because of its (typically) low processing costs, speed, repeatability, and access to macroscopic assemblies with wafer-scale or even larger macroscopic dimensions.^17−19^ Furtheremore, the fluid interface allows for manipulation of the obtained assemblies by, for example, acoustic modulation^20,21^ or alterations in the accessible surface area as it is typically achieved in a classical Langmuir trough.^22,23^ Monolayers of soft and deformable NPs confined at fluid interfaces can be compressed to a certain degree, depending on the softness and shell-to-core size ratio. Large shell-to-core ratios are achievable with precipitation and cross-linking of polymeric shells onto NPs.^24−27^ The resulting core–shell (CS) microgels are characterized by the properties of the rigid NP cores (e.g., silica, which provides enhanced optical contrast for direct monolayer visualization) and the softness and thickness of the microgel shell.^28^ When adsorbed at fluid interfaces, the microgel shells stretch laterally in the plane of the interface driven by the resulting reduction in interfacial tension.^29−31^ This effect is particularly pronounced for microgels with lower cross-linking densities, i.e., large softness,^32^ and hollow microgels with an internal cavity.^33,34^ At interparticle contact, this leads to significantly larger interparticle distances than the hydrodynamic diameter of the CS microgels measured in dispersion.^35,36^ The self-assembly of microgels and colloids, in general, at fluid interfaces is determined by the interplay between long-range attractive capillary forces, short-range attractive van der Waals forces, and repulsive electrostatic and dipolar forces.^37,38^ For CS microgels, attractive forces are significant, and cluster formation has been observed even in the dilute state.^35,36,38^ This behavior can be used to prepare freely floating monolayers of hexagonally packed microgels.^39,40^ Similarly ordered arrays of rigid spheres were prepared, and it was found that small amounts of a surfactant like sodium dodecyl sulfate (SDS) are required as a soft barrier to confine the colloid assembly.^41^ Imbalances in interfacial tension at the available surface area will further influence the monolayer behavior due to Marangoni flow.^42^ Compared with rigid spheres in the close-packed state, microgels can be compressed over a broad range of surface pressures at fluid interfaces. Therefore, imbalances in interfacial tension and the resulting Marangoni flow are expected to have a significant effect on the monolayer evolution of soft microgels. Understanding the interplay among interfacial tension, microgel structure, and the self-assembly process will offer valuable insights into the intricate dynamics governing interfacial assemblies.

In this work, the role of external interfacial tension in the time-dependent evolution of freely floating monolayers of CS microgels was investigated. To adjust the external interfacial tension, i.e., the interfacial tension of the interface surrounding the microgel monolayer, different amounts of SDS were added. An experimental procedure was developed to perform time-dependent experiments under initially equilibrated and nonequilibrated conditions. Changes in interfacial tension, total monolayer area, and local microstructure of the monolayer were monitored with time. The evolution of the monolayer area and intermicrogel distance was ascribed to Marangoni flow stemming from the interfacial tension difference between monolayer-covered and monolayer-free areas. By studying CS microgels with different cross-linker densities of their shells, we provide first insights into the role of softness in monolayer expansion. This work provides important physical insights into the assembly behavior of soft and deformable colloids at flat fluid interfaces, focusing on the role of interfacial tension imbalance. Our findings are relevant to improving our current understanding of the microgel assembly at fluid interfaces but also for the straightforward and easily implementable processes allowing for monolayer transfer with tunable interparticle distances based on low-tech and low-cost equipment.

## 2 Experimental Section

### 2.1 Chemicals

Tetraethylorthosilicate (TEOS; Sigma-Aldrich, 98%), ammonium hydroxide solution (NH_3_(aq); Sigma-Aldrich, 30–33%), ethanol (EtOH; Chemsolute, ≥99.9%), rhodamine B isothiocyanate (RITC; Sigma-Aldrich, mixed isomers), (3-aminopropyl) trimethoxysilane (APS; Sigma-Aldrich, 97%), 3-(trimethoxysilyl) propyl methacrylate (MPS; Sigma-Aldrich; 98%), sodium dodecyl sulfate (SDS; Merck; Ph. Eur.), *N*-isopropylacrylamide (NIPAM; Tokyo Chemical Industry, >98.0%), *N*,*N*′-methylenebis(acrylamide) (BIS; Sigma-Aldrich, 99%), 2,2′-azobis(isobutyronitrile) (AIBN; Aldrich, 98%), *N*,*N*-dimethylformamid (DMF; Acros, 99.8%), and potassium peroxodisulfate (PPS; Fluka; ≥99%) were used as received. Water was purified with a Milli-Q system. The final resistivity was 18 MΩ·cm.

### 2.2 Synthesis

#### 2.2.1 Synthesis and Functionalization of Silica NP Cores

To achieve fluorescently labeled silica NP cores, first, RITC was functionalized with APS. APS was added dropwise to an ethanolic RITC solution (10 mM) and stirred in the dark for at least 2 h. The amount of APS was tenfold in excess to ensure covalent binding to the dye molecule. The functionalized dye solution was diluted with ethanol in a ratio of 1:5. The silica particle synthesis was performed according to the Stöber method.^30^ A mixture containing 125 mL of EtOH and 10 mL of ammonium hydroxide solution (30–33% yield) was heated to 50 °C in a 250 mL three-necked, round-bottomed flask. The flask was equipped with a reflux condenser and a thermometer. At 50 °C, the solution was equilibrated for 20 min. Afterward, a mixture of 5 mL of TEOS and 20 mL of EtOH was heated to 50 °C and then rapidly added to the flask. When the clear solution became slightly turbid, which indicated the nucleation of silica, 2 mL of dilute solution of functionalized RITC was added dropwise. The reaction was allowed to proceed overnight at 50 °C before the final particle dispersion was cooled to room temperature. The NPs were purified and separated from the solution by centrifugation at a speed of 2599 rcf for 90 min. The supernatant was discarded, and the obtained residue was redispersed in ethanol. Then, the silica NPs were surface-modified with MPS to render these cores suitable for encapsulation by seeded precipitation polymerization. Prior to the addition of 62 μL of MPS, the pH of the silica dispersion was adjusted to 9–10 by adding an ammonium hydroxide solution (30–33%). The obtained surface density of MPS was 1 molecule per 40 Å^2^. In order to guarantee covalent binding of the MPS molecules, the mixture was stirred for 24 h and subsequently refluxed for 1 h. SDS was dissolved in 1 mL of EtOH and added dropwise to the mixture during cooling. The amount of SDS was adapted to obtain a final concentration of 0.2 mM to stabilize the silica particles. In the following, the silica particles were centrifuged at 2599 rcf for 90 min and redispersed in ethanol after discarding the clear and colorless supernatant. The purification step was repeated three times. The final particle number concentration of the silica particle dispersion is 0.193 μM determined using the density of silica and the particle volume, as calculated from the dimensions measured by electron microscopy. We used a silica density of 2.2 g/cm^3^, as reported by Masalov et al., for Stöber silica particles.^43^ The weight fraction of the dispersion was 0.155 g/mL. The monodisperse, near-spherical silica NPs had an average diameter *d*_core_ of 105 ± 6 nm, as determined by small-angle X-ray scattering (SAXS). A representative TEM image and the measured SAXS profile are provided in the Supporting Information (Figure S1).

#### 2.2.2 Seeded Precipitation Polymerization

The functionalized silica NP cores were used as seeds in free radical seeded precipitation polymerization to form PNIPAM microgel shells. To obtain three batches of CS microgels with different degrees of cross-linking, we varied the amount of BIS while keeping the amount of NIPAM constant in each synthesis (see Table 1). The respective amounts of NIPAM, BIS, and SDS (1.4 mg) were dissolved in 20 mL of water in a three-necked flask equipped with a reflux condenser. The mixture was heated to 70 °C and purged with nitrogen for 20 min to remove the oxygen. Then, the respective volume (see Table 1) of the silica seed dispersion was added. After 15 min of equilibration, 2 mg of PPS dissolved in 1 mL of water was added to the mixture to start the polymerization. The reactions were allowed to proceed for 2 h and then cooled to room temperature. The resulting CS microgels were purified by centrifugation at 2599 rcf for 90 to 180 min until we got a clear supernatant. For all CS microgels, centrifugation and redispersion in Milli-Q water were repeated three times. Finally, the CS microgels were freeze-dried. In the following, we use CS_*x*_ to refer to the respective batch of CS microgels, where *x* stands for the nominal cross-linker density in mol % with respect to the molar amount of NIPAM, i.e., 5, 10, and 15.

**Table 1:** Synthesis Conditions for the Seeded Precipitation Polymerization of Microgels CS_5_, CS_10_, and CS_15_^a^

SiO_2_–PNIPAM microgel	CS_5_	CS_10_	CS_15_
*m*(PNIPAM) [mg]	113	113	113
*m*(BIS) [mg]	8	15	23
*V*(SiO_2_) [μL]	438	480	333
*d*_core_ [nm]	105 ± 6	105 ± 6	105 ± 6
*d*_h_ (25 °C) [nm]	316 ± 4	299 ± 4	317 ± 2
*d*_h_ (55 °C) [nm]	160 ± 1	191 ± 1	218 ± 1
cross-linker density^b^[mol %]	5	10	15

a The core diameter, *d*_core_, was from SAXS, and the total hydrodynamic diameter, *d*_h_, was from dynamic light scattering (DLS) measurements.

b The values listed are nominal values and refer to the molar amount of BIS in relation to the molar amount of NIPAM in mol %.

#### 2.2.3 Synthesis of the Linear PNIPAM Homopolymer

Linear PNIPAM homopolymers were synthesized using free radical polymerization.^12^ First, 9.41 mL of DMF and 4.526 g of NIPAM were added to a three-necked flask and heated in a silicon oil bath to 70 °C. The mixture was then degassed for 30 min with argon. Then, 0.005 g of AIBN was dissolved in 0.5 mL of DMF and added to the mixture. After 3 h of polymerization, the reaction was stopped by immersing the mixture into an ice bath and exposing it to air. The products were precipitated in diethyl ether and redissolved in acetone three times. In the end, the final precipitated products were collected by centrifugation and dried under a vacuum. According to the protocol, the nominal molecular weight of the synthesized linear PNIPAM was 82 kg/mol.

### 2.3 Dynamic Light Scattering (DLS)

A Zetasizer Nano S (Malvern) was used for measuring the hydrodynamic particle dimensions as a function of the temperature. A 4 mW HeNe laser with a 633 nm wavelength was used as the light source. The scattering angle of the device was 173°. The temperature range was set between 10 and 55 °C at intervals of 1 °C. The samples were measured three times at each temperature. The data were analyzed by the cumulant method with software provided by the instrument. Hydrodynamic diameters were z-averaged values.

### 2.4 Monolayer Preparation

The spreading solution used for the monolayer preparation consisted of 1 wt % microgels, 64 wt % ethanol, and 35 wt % water. A crystallizing dish with an inner diameter of 70 mm and a PTFE ring with an inner diameter of 36.5 mm (40.5 mm outer diameter and 2.0 mm thickness) were used to prepare freely floating monolayers. First, the crystallizing dish was filled with 85 mL of water. Then, the PTFE ring was positioned at the air–water interface and left there floating. A three-dimensional (3D) printed support frame was used to fix the ring in position (see Figure S2 in the Supporting Information). Afterward, 3 μL of microgel dispersion was directly injected into the interface at the center of the PTFE ring. The microgels self-assembled at the air/water interface and formed a homogeneous monolayer restricted by the ring.

#### 2.4.1 Monolayer Manipulation by SDS

After forming a microgel monolayer in the ring, SDS solution (16 mM) was carefully injected outside the PTFE ring with volumes adjusted to yield the final concentrations of 0.1, 0.2, 0.5, or 1.0 mM, respectively. The 16 mM stock concentration was chosen to enable uniform dropwise addition while minimizing premature SDS migration into the bulk phase, ensuring precise control of the interfacial tension. Concentrations given throughout the manuscript refer to these final concentrations, considering the total volume of the respective aqueous subphase. The PTFE ring was manually removed by vertically lifting the support frame with minimal lateral motion to avoid interface agitation. For systems with SDS concentrations ≥ 0.5 mM, where stronger adhesion between the hydrophobic PTFE and interface occurred, a clean, very thin needle was gently inserted at the ring–interface contact point to assist detachment. At this time (*t* = *t*_0_), the monolayer was not confined anymore and is considered as “freely floating monolayer” throughout this work. To transfer the monolayer, a hydrophilic glass slide (1 × 1 cm^2^) was held at its edge by using tweezers and gently submerged into the water subphase at the edge of the crystallizing dish. Then, the slide was carefully moved proximal to the center of the monolayer to ensure that the monolayer’s center aligns approximately with the center of the slide upon transfer. Afterward, the slide was lifted vertically out of the water and kept upright while excess water was carefully removed from its surface using a paper towel. Finally, the monolayer was rapidly dried with a heat gun by directing airflow onto the back of the slide.^44^ Transfer was performed at different time points after removal of the PTFE ring. For each time point, the transfer procedure was repeated at least three times, and every transfer was done using different, individually prepared monolayers.

#### 2.4.2 Monolayer Manipulation by the PNIPAM Homopolymer

After forming a microgel monolayer in the ring, 170 μL of PNIPAM aqueous solution (0.001 wt %) was carefully injected outside the PTFE ring. After attaining equilibrium for 10 min, the ring was carefully removed from the interface. From this time (*t* = *t*_0_), the resulting “freely floating monolayer” was monitored by the camera of a mobile phone, and later, the monolayer area was calculated based on the recorded video. Then, for every stage of compression, 170 μL of PNIPAM solution was added outside the monolayer, followed by a 10 min equilibrium. After adding 510 μL of PNIPAM solution (in total), the central part of the monolayer was transferred to a clean glass substrate and dried with a heat gun.

### 2.5 Interfacial Tension Measurements

The tensiometer mode of Langmuir–Blodgett trough G2 (Kibron Inc., Finland) was used to measure the interfacial tension of the floating microgel monolayer and the microgel-free areas. A Wilhelmy plate (5 × 5 mm^2^) was mounted at the center of the monolayer or in the microgel-free areas. The interfacial tension was measured every 0.2 s during the time-dependent monolayer experiments.

### 2.6 Determination of the Monolayer Area

The time-dependent evolution of freely floating monolayers was monitored by the camera of a mobile phone, leading to videos with a resolution of 1080 pixels × 2240 pixels. The camera was fixed just above the monolayer. The pixel-to-real ratio was calibrated by the inner diameter of the crystallizing dish. The processing of the videos and the determination of the monolayer area at certain time points were performed using the ImageJ program.

### 2.7 Atomic Force Microscopy (AFM)

The microstructure of the transferred monolayers on glass substrates was investigated by AFM using a NanoWizard 4 (JPK Instruments, Germany). Height profiles were recorded from the central parts of the monolayers using intermittent contact (AC) mode with a 0.5 Hz scan rate. Images of dimensions 10 × 10 μm^2^ were recorded, acquiring 1024 × 1024 pixels^2^. The measured height profiles were flattened (first order) using Data Process Analysis to correct the tilt of the sample.

### 2.8 Drop Shape Analysis

A drop shape analyzer DSA25 (Krüss, Germany) was used to measure the interfacial tension of aqueous dispersions of the CS microgels. A drop of microgel solution was formed inside a rectangular, transparent cuvette (1 × 1 × 4 cm^3^) to slow the evaporation of the droplet. The volume of the droplet was set around 15 μL. The interfacial tension was measured until the microgels fully absorbed and reached a saturated state at the interface.

## 3 Results and Discussion

**Figure 1:**
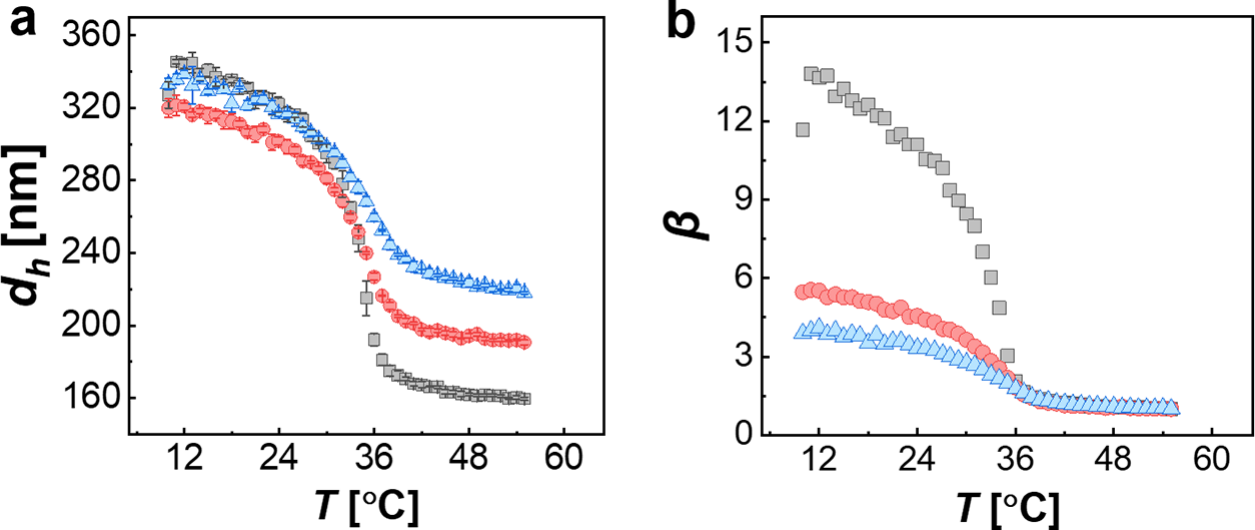
Results from temperature-dependent DLS measurements for the different cross-linked microgels: CS_5_ (gray squares), CS_10_ (red circles), and CS_15_ (blue triangles). (a) Hydrodynamic diameters, *d*_h_, as a function of the temperature. The error bars correspond to the standard deviation from three measurements. (b) Corresponding swelling ratios β.

We prepared three different SiO_2_–PNIPAM CS microgels by seeded precipitation polymerization using SiO_2_ cores from the same batch. This means that all CS microgels have the same average core size and size distribution (105 ± 6 nm, as determined by SAXS). To facilitate comparison, we aimed at CS microgels that feature similar shell thicknesses in the swollen state, i.e., at 25 °C (see Table 1), but differ in cross-linker density, i.e., softness of the shell. The difference in cross-linking can be confirmed by studying the volume phase transition (VPT) of the CS microgels.^45^Figure 1a shows the swelling curves in terms of hydrodynamic diameters, *d*_h_, as a function of the temperature obtained from DLS. All three CS microgels show the typical VPT behavior known from PNIPAM microgels in water. In the studied temperature window, we observed a continuous decrease in *d*_h_ with increasing temperature, with the strongest decrease at the VPT temperature, which is approximately 34–35 °C for the three microgels. The difference in swelling capacity can be best expressed when comparing the relative change in hydrodynamic volume with respect to a reference state. Figure 1b shows the temperature evolution of swelling ratio β. To calculate β, the volume of the nonswellable SiO_2_ cores was subtracted
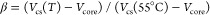
1Here, *V*_cs_(*T*) corresponds to the hydrodynamic volume of the CS microgels at temperature *T*, *V*_core_ is the volume of the SiO_2_ core based on the diameter from SAXS measurements, and *V*_cs_(55 °C) is the volume of the CS microgel in its fully collapsed state at 55 °C. The swelling ratios β show that, starting from the collapsed as the reference state, the CS_5_ microgels increase in volume by a factor of approximately 14 when the temperature is reduced below the VPTT. In contrast, the highest cross-linked CS_15_ microgels show an increase in volume by a factor of only approximately 4. We now start with results obtained from the CS_10_ microgels as representative microgels with intermediate cross-linking. The difference in swelling capacity will become relevant later in the article when we directly compare monolayers of the three CS microgels.

### 3.1 Freely Floating Monolayers at Flat Air/Water Interfaces

**Figure 2:**
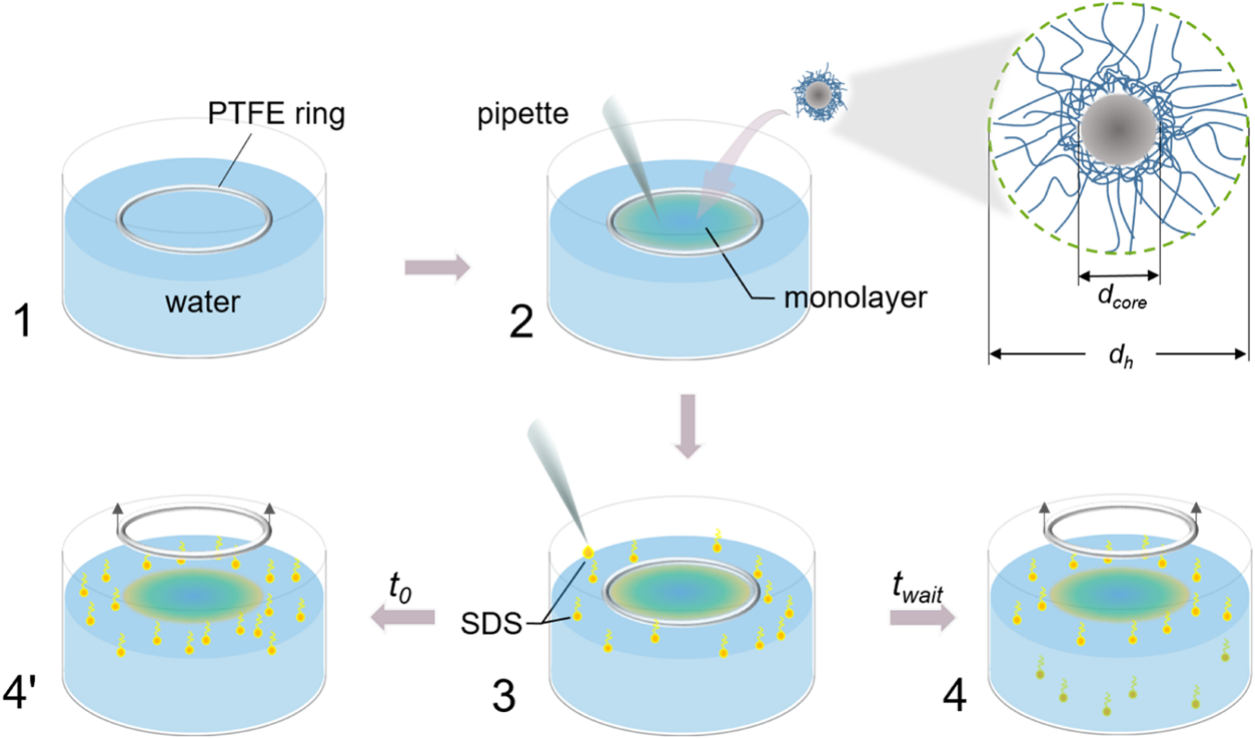
Schematic diagram illustrating the preparation of a floating monolayer of CS microgels with a core diameter, *d*_core_, and overall hydrodynamic diameter, *d*_h_, at air/water interfaces: (1) a crystallizing dish was filled with water, and a PTFE ring was positioned at the air/water interface; (2) upon injection of the microgel dispersion directly to the interface using a pipette, the forming monolayer was first restricted by the PTFE ring; (3) then, SDS was injected to adjust the interfacial tension outside the PTFE ring; and finally, the PTFE ring was removed (4′) immediately or (4) after some equilibration time.

Freely floating monolayers of CS microgels can be prepared by simply injecting a spreading solution containing the microgels directly at the air/water interface.^39^ In the present case, our spreading solution consists of a mixture of 64 wt % ethanol, 35 wt % water, and 1 wt % microgels, which acts as a solvent for the microgels and facilitates their smooth spreading at the air/water interface due to Marangoni flow.^20^ In order to study the time-dependent evolution of such monolayers, we need to define reference conditions for the start of the experiment, i.e., at time *t* = *t*_0_. To do so, we developed a simple protocol that (1) defines the monolayer area in the reference state and (2) defines *t* = *t*_0_. This procedure is schematically depicted in Figure 2.

**Figure 3:**
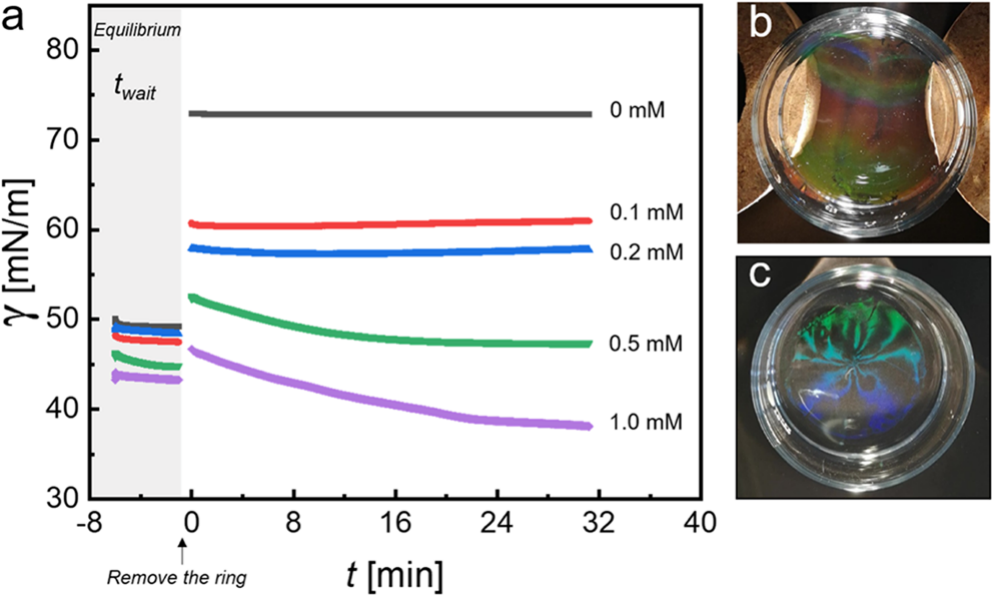
Time-dependent evolution of the interfacial tension, γ, measured within the monolayer-covered (CS_10_ microgels) area for different SDS concentrations. (a) Interfacial tension as a function of time prior to (gray background) and after removal of the PTFE ring at *t* = 0 min. The SDS concentrations used to adjust γ outside the monolayer-covered areas were 0, 0.1, 0.2, 0.5, and 1.0 mM, respectively. Photographs of the freely floating monolayer right after removal of the PTFE ring for (b) 0 mM and (c) 0.1 mM SDS.

Upon filling a crystallizing dish with the desired volume of water (bulk subphase), we placed a PTFE ring at the air/water interface (1) with the help of a 3D printed support frame (shown in Figure S2 in the Supporting Information). (2) Then, we injected the spreading solution containing the CS microgels directly into the interface inside the PTFE ring. During the injection, a monolayer of microgels is formed immediately, filling up the available interfacial area. This strategy for confinement of a colloidal monolayer at fluid interfaces is similar to the one reported by Lotito et al.^46^ Depending on the volume of the injected solution, the area inside the ring could be completely filled with the monolayer. After microgel deposition to the interface, we injected a 16 mM SDS stock solution outside the ring dropwise. SDS was chosen because it is a well-studied “standard” surfactant that has been used in the literature to laterally confine monolayers of rigid colloids^41^ and has also been employed to prepare highly ordered microgel monolayers.^39^ All SDS concentrations in this work (0.1–1.0 mM) are below the critical micelle concentration (CMC ≈ 8 mM), ensuring that interfacial tension reduction arises solely from monomer adsorption.^47^ This avoids confounding effects from micelle formation, which could alter the dynamics of Marangoni flow. This way, we could adjust the interfacial tension outside the PTFE ring by adjusting the amount of the injected SDS solution. After the PTFE ring was removed, we obtained a freely floating monolayer. The time of the removal of the ring defined the start of the experiment, i.e., *t* = *t*_0_. In the following, we discuss two different sets of experiments performed this way: (1) equilibrium experiments, where we waited long enough until a constant interfacial tension was measured outside the PTFE ring, and (2) nonequilibrium experiments, where we removed the ring immediately after the SDS injection. We first discuss the equilibrium experiments. Figure 3 shows the time-dependent evolution of the interfacial tension, γ, measured within the microgel monolayer for different SDS concentrations, i.e., values of γ outside the monolayer.

For each of these individual experiments, we injected the same volume of microgel dispersion into the interface available inside the PTFE ring. Thereby, we made sure that the experiments were comparable. We start with very similar numbers of microgels per area, *N*_p_/*A*. The corresponding targeted initial interfacial tensions were approximately 49.0 mN/m. To guarantee that equilibrium was reached and stable values of γ were obtained outside the monolayer, we waited for 60 min (*t*_wait_ = 60 min) after injection of SDS to the outside of the PTFE ring prior to its removal. The stability of the monolayer within the ring is challenging to sustain beyond 60 min when the SDS concentration is higher than 0.5 mM. This suggests that at higher SDS concentrations, a small amount of SDS might migrate into the ring through the subphase, disrupting the integrity of the monolayer. In Figure 3a, we see that, starting from *t* = −5 min, i.e., prior to the removal of the PTFE ring, initial values of γ decrease with increasing SDS concentration. For 0 mM SDS, the interfacial tension remained at a constant value of 49 mN/m, which corresponds to the initially targeted value. For the highest SDS concentration of 1.0 mM, the γ within the monolayer-covered area was measured as 43.2 mN/m. This decrease of almost 6 mN/m is related to SDS adsorption to the interface within the microgel-monolayer-covered area during the equilibration time. Since this adsorption happened already before the removal of the ring, some mass transport must have occurred via the bulk subphase.

Since the ring largely blocked mass transport to the monolayer, this procedure allows us to adjust the difference of interfacial tension Δγ inside and outside of the monolayer. After removal of the ring, depending on the sign of Δγ, we observed either expansion or shrinkage of the monolayer until Δγ ≈ 0 mN/m. When the SDS concentration was 0, 0.1, or 0.2 mM, the interfacial tension values outside the monolayer were larger than 49.0 mN/m and monolayers expanded outward after removing the ring. These changes in the monolayer area happened instantaneously. As a consequence of the expansion, the interfacial tension within the monolayer increased abruptly to 73.9, 60.7, and 58.0 mN/m, respectively. These values remained nearly constant during the course of the experiments. In comparison, the expansion measured in a Langmuir–Blodgett trough occurs within an interfacial tension range of 43.0–70.5 mN/m (see Figure S3 in the Supporting Information). When the SDS concentration was 0.5 mM, corresponding to a small Δγ, the value of γ abruptly increased to 52.6 mN/m after removal of the PTFE ring and then slowly decreased to 47.4 mN/m. For 1.0 mM SDS, the initial equilibrium interfacial tension outside the monolayer is lower than that within the monolayer. In this case, upon removal of the PTFE ring, γ first abruptly increased to 46.7 mN/m and then slowly decreased to a final equilibrium value of 38.0 mN/m. The first abrupt increase in interfacial tension for 0.5 and 1.0 mM SDS is attributed to the removal of the PTFE ring and the newly generated air–water interface with large interfacial tension in the area initially covered by the ring. In contrast to the monolayer expansion for lower SDS concentrations, the reported monolayer compression is time-dependent, involving slow relaxation processes probably related to local rearrangements.

We monitored the freely floating monolayers with a digital camera placed above the experimental setup during these experiments. Figure 3b,c shows the photographs of the monolayer right after removal of the PTFE ring for 0 and 0.1 mM SDS, exemplarily. The monolayer is clearly visible due to the iridescence caused by the periodic arrangement of microgels with interparticle distances on the order of the visible wavelength. Comparing the two photographs, we see a difference in the iridescence color with a reddish/green coloration for the experiment at 0 mM SDS and a greenish/blue coloration for 0.1 mM SDS. This difference in coloration is attributed to the different overall monolayer dimensions and, due to the very similar particle numbers in the monolayers, the difference in interparticle spacing. In the absence of SDS, the monolayer uniformly spreads across the entire available area in the crystallizing dish. However, when a concentration of 0.1 mM SDS is present, the monolayer covers a reduced area, resulting in shorter interparticle distances and consequently a more pronounced, blue-shifted iridescence. We can conclude from the data of Figure 3 that imbalances between the interfacial tension outside and inside the monolayer-covered area induce the expansion or compression of the freely floating monolayer. Table 2 summarizes the observations from these experiments and the relevant interfacial tension values.

**Table 2:** Parameters and Observations for CS_10_ Monolayer Experiments in Dependence of SDS Concentration, *c* (SDS)^a^

*c* (SDS) [mM]	γ_*t*=0_ (SDS)^b^ [mN/m]	γ_*t*=0_ (ML)^a^ [mN/m]	observation	γ_*t*=32_ (ML) [mN/m]
0.0	72.9	50.0	expansion	72.8
0.1	64.1	48.2	expansion	61.0
0.2	60.4	48.8	expansion	57.7
0.5	56.8	45.8	stagnation	47.4
1.0	50.4	43.2	compression	38

a Initial interfacial tension at equilibrium measured outside the PTFE ring, γ_*t*=0_ (SDS), initial interfacial tension of the monolayer at *t* = 0 min, γ_*t*=0_ (ML), and interfacial tension values of the monolayer, γ_*t*=32_ (ML), measured after 32 min.

b Values were measured using a Wilhelmy plate after 60 min of equilibration time after the addition of SDS to the outside of the PTFE ring.

**Figure 4:**
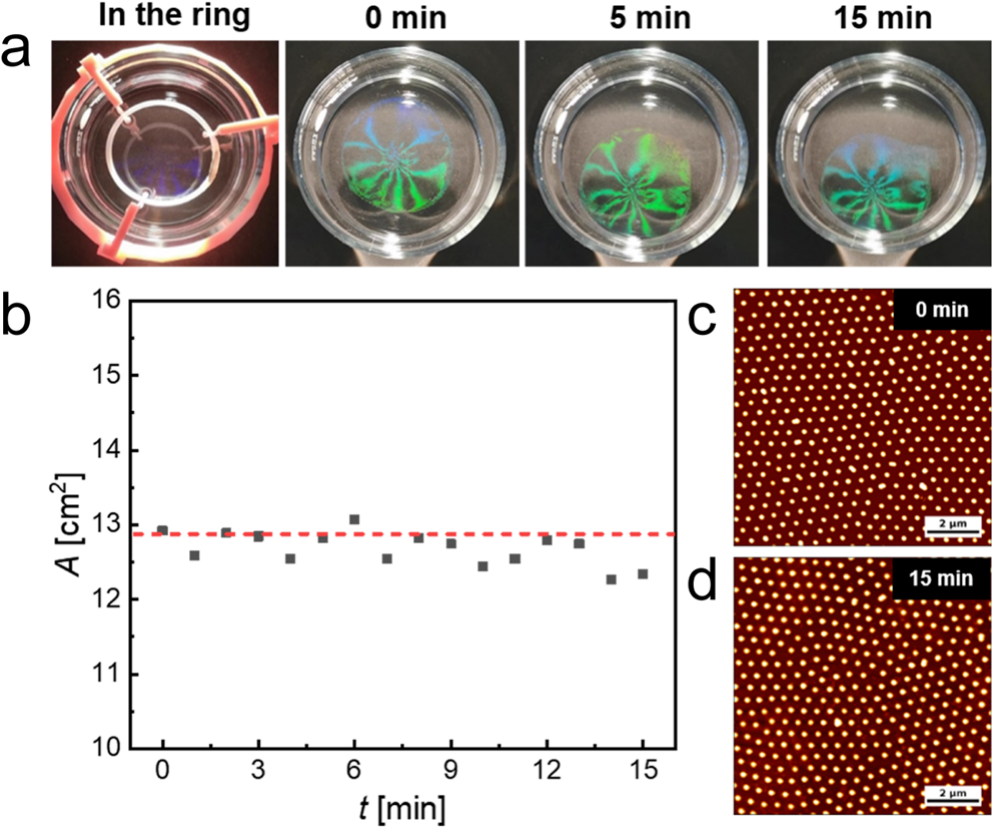
Time-dependent evolution of the CS_10_ microgel monolayer for 0.5 mM SDS at an equilibrium state (120 min equilibration time after SDS addition). (a) Photographs of the monolayer before and after different times of removal of the PTFE ring. (b) Time-dependent evolution of the monolayer area, *A*. The horizontal, red dotted line indicates the initial available area defined by the PTFE ring (based on the outer radius). AFM height profile micrographs recorded from substrate-supported samples (ex situ) withdrawn after (c) 0 and (d) 15 min.

In the case of 0.5 mM SDS, Δγ is small, and we hypothesize that in this case the monolayer remains rather unchanged after removal of the PTFE ring. To confirm this hypothesis, we followed the time-dependent evolution of the monolayer area for 0.5 mM SDS and direct AFM-based quantification of *d*_c-c_ (Figure 4c,d). Figure 4a shows the snapshots recorded at different times in the experiment. Again, the monolayer and its area can be distinguished due to its iridescence. Within the PTFE ring, the confined CS microgel monolayer shows a purple-bluish color that is more difficult to see. In this starting scenario, the diffraction color, as captured by the camera, is at the lower wavelength end of the visible spectrum, indicating relatively small interparticle distances at the initial stage of the experiments. At *t* = 0 min, i.e., immediately after removal of the PTFE ring, the monolayer could be easily identified with a green/blue structural color. Critically, while the color shift (e.g., purple to green) qualitatively reflects an increase in interparticle spacing over time, optical diffraction was not used to calculate the *d*_c-c_ values. Photographs taken at later times, i.e., at 5 and 15 min after removal of the PTFE ring, reveal freely floating monolayers of similar total dimensions and structural colors. To ensure measurement reliability, we limited the experimental duration to 15 min, as the central region remained stable within this time frame, whereas peripheral instability—characterized by increased interparticle distances and reduced ordering, as reported by Volk et al.^39^—led to partial edge detachment beyond it (Figures 4 and S5). Figure 4b shows the detected total monolayer area, *A*, as a function of the time starting right after removal of the PTFE ring. Over the course of the experiment, *A* was found to exhibit only a slight decrease that could relate to the slight decrease of the interfacial tension observed in Figure 3a.

Figure 4c,d shows the AFM height images recorded from monolayer samples that were collected at the beginning and end of the experiment. The microstructure in both images is very similar, and the nearest-neighbor center-to-center distances, *d*_c-c_, were determined at 469 ± 28 and 496 ± 31 nm for 0 and 15 min, respectively. Note that these distances are largely greater than the hydrodynamic diameter of the CS microgels pointing to a “corona–corona” interaction throughout the whole expansion process. The initial *d*_c-c_ of the monolayer (before removal of the ring) was 438 ± 12 nm. This slight increase in *d*_c-c_ (approximately 10%) is on the order of the diameter that was initially occupied by the PTFE ring. i.e., the difference in area of the ring calculated using its inner and outer radii. That is to say, the microgels immediately filled the free interface formerly occupied by the ring. It is worth noting that the AFM micrographs capture only very small areas and thus small numbers of CS microgels, representing the local microstructure. The differences in *d*_c-c_ at the beginning and end of the experiment might point to local variations in microstructure (e.g., degree of order). Nevertheless, the combined data of Figure 4 reveal either stagnation or a very slight compression of the freely floating monolayer when the interfacial tension imbalances are very small. In summary, the equilibrium procedure allows for the creation of a stable monolayer with a desired area by adjusting the SDS addition. However, when aiming to investigate the mechanism of monolayer expansion, an alternative procedure is necessary—one that is slow enough to facilitate the monitoring of the expansion process.

### 3.2 Equilibrium vs Nonequilibrium Starting Conditions

**Figure 5:**
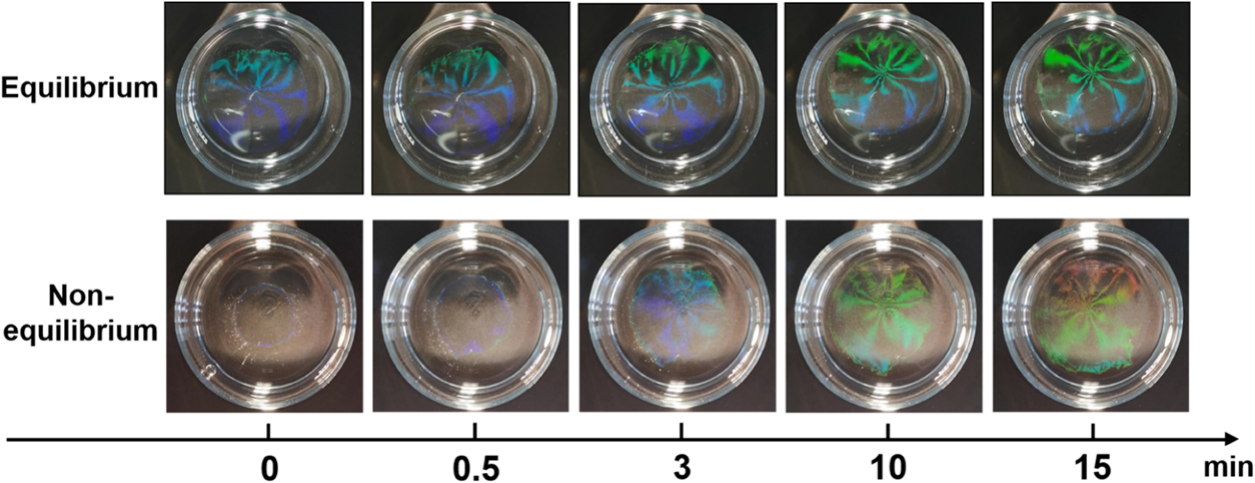
Digital photographs of freely floating CS_10_ monolayers taken at different time intervals after removal of the PTFE ring. The concentration of SDS was 0.1 mM in these experiments. The images in the top row correspond to the equilibrium state after 60 min of equilibration, i.e., when the interfacial tension outside the monolayer had stabilized after the addition of SDS. The images in the bottom row correspond to a nonequilibrium experiment, where the PTFE ring was removed right after the addition of SDS. The photographs show the change of the monolayer area with SDS equilibrated for 60 min (top row) and immediately after SDS addition (nonequilibrium, bottom row).

Figure 5 shows the photographs of freely floating CS_10_ monolayers at different time intervals after the removal of the PTFE ring for 0.1 mM SDS, i.e., conditions under which the monolayer is expected to expand. The photographs in the top row correspond to the case where we waited for 60 min before removing the ring after SDS addition. After the ring was removed, the monolayer instantaneously expanded at the interface and then remained basically unchanged for a long period of time (Figure 5, top row). Under such conditions, the initial expansion of the freely floating monolayers caused by the significant interfacial tension imbalance occurred very fast—too fast to be monitored in our experiment. The interfacial tension of the monolayer within the ring was measured to be 49 mN/m with a corresponding monolayer area of 10.5 cm^2^, as determined by the inner radius of the ring. Subsequently, upon removal of the ring, the monolayer area expanded immediately to 17.8 cm^2^ and remained nearly constant over the course of the experiment (Figure 5, top row). At later times, the area of the monolayer is not easily trackable. The associated interfacial tension of the expanded monolayer was measured at 66 mN/m.

**Figure 6:**
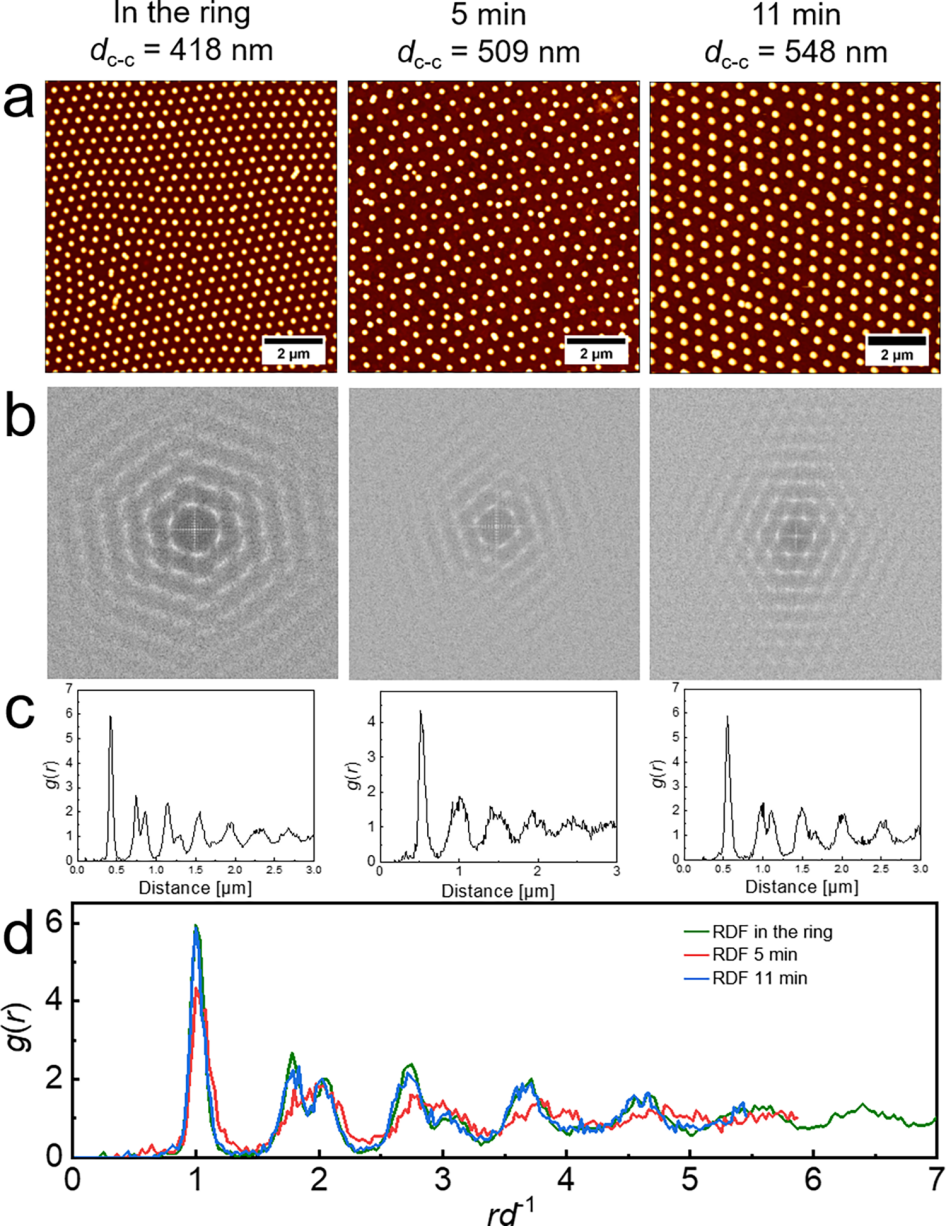
Structural characterization of CS_10_ monolayers after transfer to glass substrates for an expansion experiment at 0.1 mM SDS. (a) AFM height profiles of the monolayers transferred from the air/water interface when confined inside the PTFE ring and 5 and 11 min after removal of the PTFE ring. (b) Fast Fourier transformations (FFTs) computed from the corresponding AFM images shown in (a). (c) Radial distribution functions, *g*(*r*), computed from the point maps of the AFM images. (d) Radial distribution functions normalized to the nearest-neighbor center-to-center distance of the monolayers.

**Figure 7:**
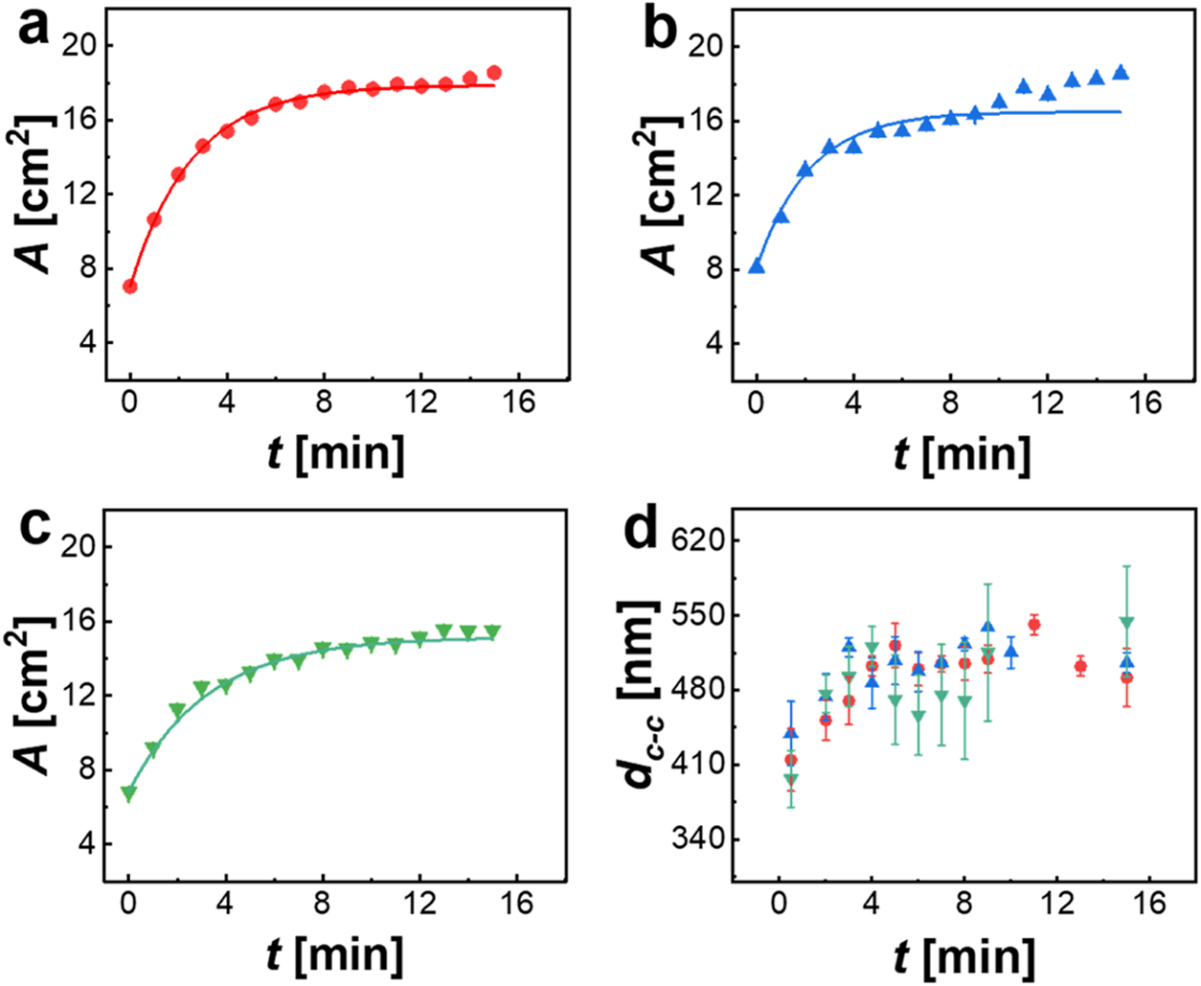
Expansion kinetics of monolayers of CS_10_ microgels at the air/water interface for SDS concentrations of 0.1 mM (red circles), 0.2 mM (blue triangles), and 0.5 mM (green triangles). (a–c) Measured monolayer area *A* (symbol) and fits to the data according to eq 2 as solid lines. (d) Evolution of *d*_c-c_ as a function of time obtained from ex situ analysis by AFM of monolayers transferred to glass substrates.

In stark contrast, when the PTFE ring was removed right after the addition of SDS to the outside of the PTFE ring (nonequilibrium), the monolayer first rapidly compressed to a smaller size in the beginning (*t* = 0 min) and then expanded with increasing time (Figure 5, bottom row). The corresponding quantitative evolution of the monolayer area over time is plotted in Figure 7a and will be discussed at a later stage. To follow the evolution of the monolayer microstructure during expansion under nonequilibrium conditions, we transferred monolayer samples to solid substrates at various time intervals after the removal of the ring. The resulting substrate-supported monolayer samples were then investigated by AFM. Figure 6 shows the AFM height profiles of monolayers transferred from inside the PTFE ring and 5 and 11 min after the addition of 0.1 mM SDS and subsequent removal of the ring. The images reveal that the monolayer consists of microgels self-assembled into a homogeneous hexagonal lattice with a *d*_c-c_ of 418 nm. With increasing time after removal of the PTFE ring, *d*_c-c_ increased to 509 nm after 5 min and 548 nm after 11 min. At the same time, the hexagonal order of the arranged microgels was maintained, as shown by the large number of Bragg peaks in the fast Fourier transformations (FFTs) in Figure 6b. The hexagonal order and the pronounced spatial correlation over several microgel diameters are further confirmed by the computed pair correlation functions (*g*(*r*)) in Figure 6c,d. The *g*(*r*)s normalized with respect to their position of the first peak shown in Figure 6d further indicates that the structural arrangement during the expansion is accompanied by a slight loss of structural ordering that is recovered after 11 min. In summary, the nonequilibrium procedure enables us to transfer the monolayer at different time points, allowing for the observation and investigation of the expansion process and the subsequent rearrangement of the monolayer microstructure.

### 3.3 Monolayer Expansion at Different External Interfacial Tensions

To further investigate the expansion behavior of monolayers for different SDS concentrations under nonequilibrium conditions, the change of the monolayer area was monitored macroscopically by video recording from the top. Figure 7a–c show the time evolution of the total monolayer areas obtained from image analysis. For all three SDS concentrations, the monolayer areas at *t* = 0 min are on the order of 7–8 cm^2^. This value is significantly lower than the initial monolayer area (10.5 cm^2^) defined by the area inside the PTFE ring prior to the start of the time-dependent experiment. The immediate shrinkage observed in nonequilibrium experiments (e.g., 0.1 mM SDS, Figure 5, bottom row) is attributed to a rapid imbalance in interfacial tension upon the removal of the PTFE ring. Before SDS reaches equilibrium, the surrounding interface exhibits a significantly lower interfacial tension than the monolayer-covered region, inducing the initial monolayer shrinkage. This compression, occurring on time scales too short to be directly monitored, leads to an initial decrease in the monolayer area. Since the SDS stock solution was added onto the surface and its subsequent diffusion throughout the bulk phase is a time-dependent process, there was a large surface excess concentration right at the beginning of the experiments. Desorption of the SDS excess takes place during the experiments, leading to an increase of interfacial tension in the monolayer-free area and an increase in SDS concentration in the bulk subphase. This is accompanied by a continuous increase in the monolayer area, *A*(*t*), with time until approaching nearly constant values at the end of the experiments (15 min). The final value for 0.5 mM closely matches the values reported in Figure 3b, indicating that a similar final equilibrium state is reached. The final values of *A*(*t*) for 0.1 and 0.2 mM SDS are larger than the area initially defined by the PTFE ring. This means that both monolayers at these low SDS concentrations expanded with respect to the initial state. The solid lines in Figure 7a–c correspond to fits according to
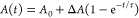
2Here, *A* is the monolayer area, *t* is the expansion time, *A*_0_ is *A* at *t* = 0 min, τ is the relaxation time, and Δ*A* is the area difference between *A*_0_ and *A* at *t* = 15 min. This model closely resembles the viscoelastic relaxation of a Kelvin–Voigt material subjected to sudden stress. In particular, for the lowest and highest SDS concentrations, the agreement between data and fit is very good. Table 3 lists the results from the fits to the data.

**Table 3:** Results from Fits to Time-Dependent Data (Figure 7) are Presented Using eq 2

*c* (SDS) [mM]	*A*_0_ [cm^2^]	Δ*A* [cm^2^]	τ [min]
0.1	7.04	10.85 ± 0.04	2.54 ± 0.04
0.2	8.11	8.41 ± 0.08	2.18 ± 0.19
0.5	6.84	8.33 ± 0.16	3.28 ± 0.18

As expected, the total change in monolayer area, Δ*A*, increases with decreasing SDS concentration, i.e., increasing Δγ. The relaxation time, τ, is largest for 0.5 mM SDS and significantly decreased for the lower SDS concentrations, indicating faster expansion of the monolayers for higher Δγ that enhances the Marangoni flow. The trend in relaxation times, however, is not that clear, which we attribute to the less good fit to the data for 0.2 mM, which leads to the most unreliable value of τ (2.18 min).

**Figure 8:**
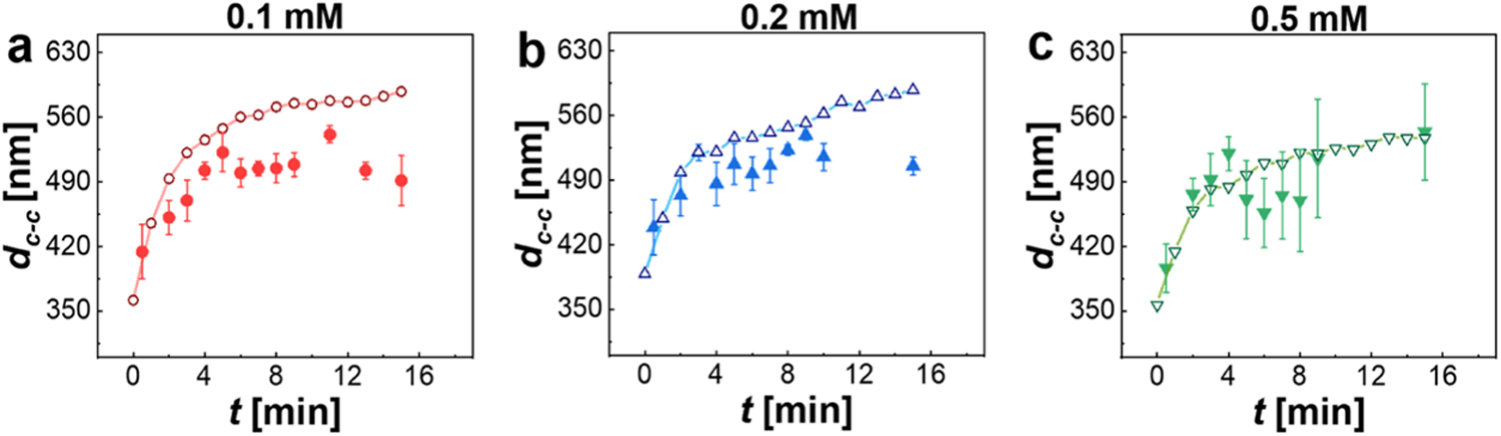
Development of *d*_c-c_ of CS_10_ monolayers over time for three different SDS concentrations of 0.1 mM (a), 0.2 mM (b), and 0.5 mM (c). The filled symbols correspond to experimentally measured values from microstructural analysis by AFM at given points in time. The open symbols and the corresponding guide-to-the-eye lines correspond to theoretically calculated values from the measured total monolayer areas, *A*(*t*), assuming hexagonal packing of the microgels in the monolayer.

To establish a connection to local length scales, the central parts of the monolayers were transferred to glass substrates and imaged by AFM after drying. The time-dependent evolution of *d*_c-c_ was extracted from image analysis. Figure 7d shows a continuous increase in *d*_c-c_ for all three SDS concentrations, starting with values of approximately 410 nm. Since the values at each time show relatively large standard deviations, we cannot extract a clear correlation of the local microstructural changes to the SDS concentration. We want to highlight that rather small monolayer areas were probed by AFM, leading to larger uncertainties. In addition, the transfer protocol to the solid substrates and the monolayer drying may have affected the microstructure in terms of the degree of order and spatial arrangement—at least locally compared to much larger CS microgels where significant changes were observed depending on microgel softness and substrate wettability.^36^ In addition, it is also possible that the kinetics of the monolayer expansion vary on the periphery of the monolayer compared to its center. To conclude, we observed expansion of the monolayer from a macroscopic (*A*(*t*)) and local viewpoint (*d*_c-c_(*t*)) for all three nonequilibrium experiments. To further support this correlation, we plotted the time-dependent evolution of *d*_c-c_ in direct comparison to the theoretically calculated values based on the change in *A*(*t*), as shown in Figure 8. The theoretical values were calculated under the assumption that the monolayer with its area *A*(*t*) contains a constant number of microgels during the expansion and maintains a perfect hexagonal arrangement with an area fraction of 0.91. The following equations were used to calculate the theoretical nearest-neighbor distances
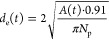
3
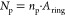
4Here, *d*_*e*_(*t*) is the theoretical *d*_c-c_(*t*), *A*(*t*) is the monolayer area, *N*_p_ is the number of microgels in the monolayer, and 0.91 corresponds to the maximum area fraction for hexagonal packing of circles in 2D. Note that this calculation does not account for the possible microgel faceting at high monolayer compression. The number of microgels in the monolayer was calculated using eq 4. Here, *n*_p_ is the number of microgels in a 10 × 10 μm^2^ area of the monolayer in the ring, which was determined by AFM analysis, and *A*_ring_ is the area of the PTFE ring calculated using the inner radius.

We see that the experimental values closely follow the theoretical expectation, and significant deviations are only observed for 0.1 and 0.2 mM SDS at longer times. In these two cases, the experimental values are smaller than those derived from the local microstructures. This might point to (1) lower degrees of order in the monolayer samples and/or (2) potential drying effects where immersion capillary forces reduce *d*_c-c_. Specifically, drying effects during transfer to a solid substrate—such as capillary-driven transitions and localized shell deformations—can distort the interfacial microstructures, as demonstrated in our prior studies.^35,36^

Furthermore, since the expansion of the monolayer is driven from the outside of the monolayer, we expect that local changes in *d*_c-c_ are larger in the outside region of the monolayer compared to its central region. This would imply a radial gradient in *d*_c-c_ values with smaller values at the center of the monolayer and larger values on the periphery. In addition, the shape of the monolayer, the perturbation of the interface when removing the ring, and the drifting of the monolayer could also induce variations in the microstructure, which would result in different values of *d*_c-c_ at different positions. Nevertheless, we clearly see that the macroscopic expansion of the total monolayer is reflected by local changes in the interparticle spacing. This means that we can simply use interfacial tension to tailor the lattice parameter in crystalline microgel monolayers, which is interesting, e.g., for photonic and lasing applications.

### 3.4 Influence of Cross-Linker Density on Monolayer Expansion

We now want to compare the time-dependent evolution of the freely floating monolayer of CS microgels in dependence of the cross-linker density, i.e., the softness of the microgels.^32^ To do so, we prepared monolayers of CS microgels with 5, 10, and 15 mol % cross-linker densities (nominal), aiming for a similar *N*_p_/*A* by injecting the same volume of CS microgel stock dispersion with the same solid content. Initially, these monolayers were confined by the PTFE ring that also defines the monolayer area, *A*_0_, at time *t* = 0 min. Upon injection of SDS (0.1 mM) outside of the PTFE ring, the PTFE ring was removed, and the monolayer evolution was monitored (see Figure 2).

**Figure 9:**
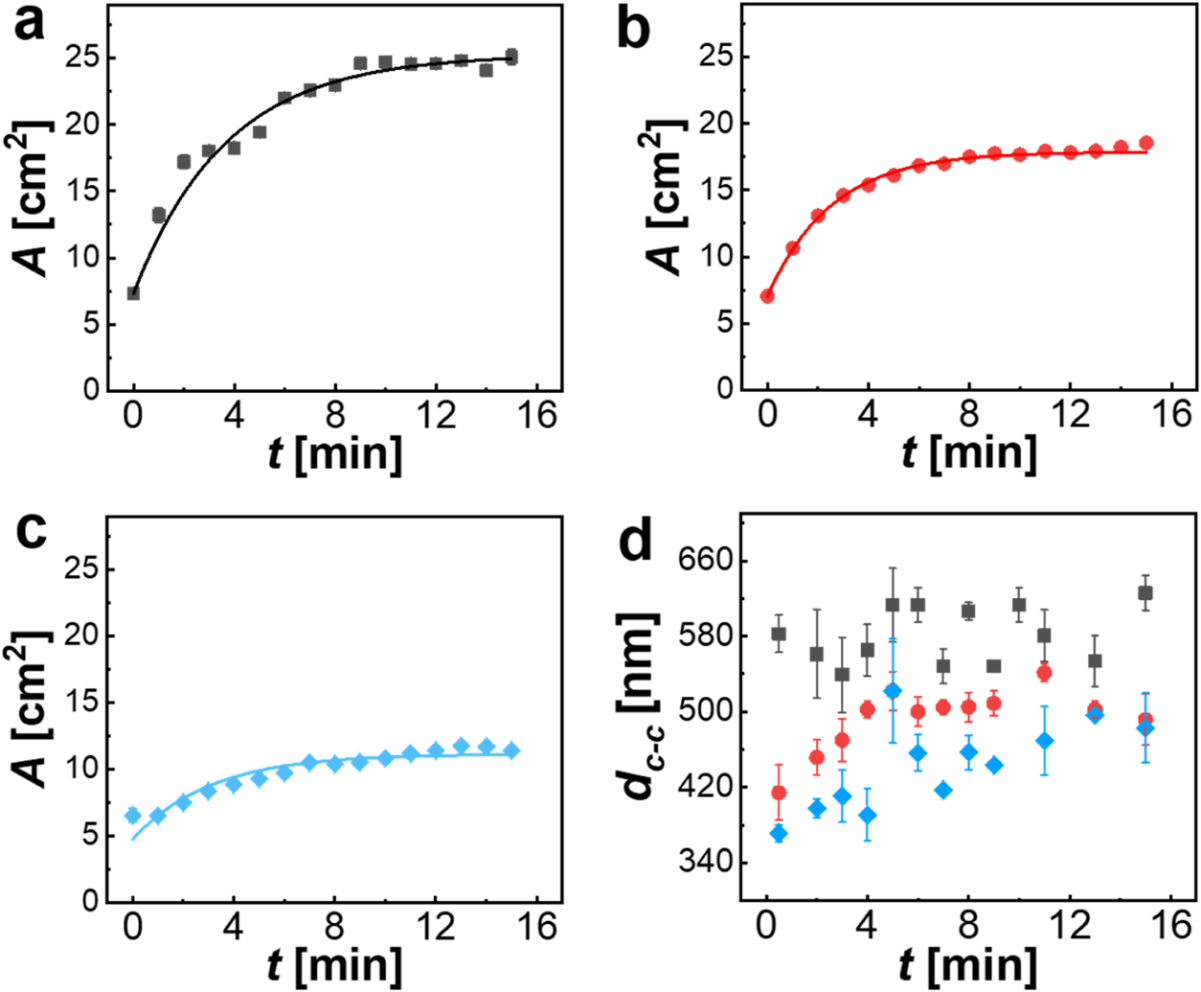
Influence of cross-linker density on monolayer expansion for an SDS concentration of 0.1 mM. Black squares correspond to 5 mol % (CS_5_), red circles correspond to 10 mol % (CS_10_), and blue diamonds correspond to 15 mol % (CS_15_) cross-linking. (a)−(c) Measured monolayer area *A* (symbol) and fits to the data according to eq 2 as solid lines. (d) Evolution of *d*_c-c_ as a function of time.

Figure 9 shows that for all cross-linker densities, the monolayer area, *A*, increased during approximately 10 min of the experiments and afterward attained rather constant values. The extent of expansion, however, strongly depends on the cross-linking. With respect to the initial area (*t* = 0 min), the monolayers increased in area by 243% (CS_5_), 164% (CS_10_), and 76% (CS_15_) within 15 min. We monitored the time-dependent evolution of the local microstructure by transferring samples from the central region of the monolayer to solid substrates and then imaging the dried monolayers by AFM. Figure 9d shows the resulting time-dependent evolution of *d*_c-c_ for the three CS microgels. We see a clear and significant increase in *d*_c-c_ over time for the medium and highly cross-linked microgels, although the trend is not as clear as in Figure 9a–c, and the standard deviations are large. For the lowest cross-linked CS_5_ microgels, the values of *d*_c-c_ do not show a clear trend with time. This unexpected result might be related to the previously mentioned changes during monolayer drying, the rather poor statistics of the applied analysis of *d*_c-c_—at least in comparison to the large, macroscopic size of the total monolayer—and the potential distribution of interparticle spacings from the center toward the outside of the monolayer. However, our findings also point to a difference between low and high cross-linked microgels. To further elaborate on this potential influence, further experiments and, in particular, support from theoretical simulations will be needed in a future study.

Once again, the data could be successfully fitted using the model depicted by eq 2. The fit results are summarized in Table 4. Considering that all three experiments were performed with the same amount of SDS and assuming that the desorption kinetics should be comparable, we attribute differences in τ to be mostly related to the different moduli *E* of the microgels. We found the largest value of τ for the lowest cross-linked microgels, pointing to the lowest value of *E*. However, it is noteworthy that the τ value for CS_15_ is unexpectedly larger than the value for CS_10_. The total changes in the monolayer area, Δ*A*, however, confirm the expected differences in microgel softness, with the minimum change in the area for the CS_15_ microgels, i.e., the microgels with the least deformable shell, i.e., the lowest swelling ratio β (see Figure 1b).

**Table 4:** Parameters Obtained from Fits (eq 2) to the Data Shown in Figure 9 for the CS_5_, CS_10_, and CS_15_ Monolayers for 0.1 mM SDS

microgel	a*A*_0_ [cm^2^]	Δ*A* [cm^2^]	τ [min]
CS_5_	7.32	17.95 ± 0.15	3.68 ± 0.10
CS_10_	7.04	10.85 ± 0.04	2.54 ± 0.04
CS_15_	4.74 ± 0.55	6.41 ± 0.52	3.07 ± 0.24

a For the fits, *A*_0_ values for CS_5_ and CS_10_ monolayers were fixed using the experimental values measured at *t* = 0 min, while the *A*_0_ value for the CS_15_ monolayer is the result obtained by fitting with *A*_0_ as a variable due to its lower visibility in the early stages, leading to larger uncertainties in the experimentally measured monolayer areas at shortest time intervals.

### 3.5 Manipulation of Monolayers through the Linear PNIPAM Homopolymer

In the previous section, we have shown how the interfacial tension imbalance influences the behavior of microgel monolayers, i.e., expansion and compression, depending on the sign of the interfacial tension difference. We have controlled this through the addition of SDS that has a significantly different adsorption energy from the used CS microgels. We observed strong desorption from the interface when an excess of SDS was purposely added to the surrounding interface. We also found an indication that SDS can migrate into the microgel monolayer via diffusion from the bulk subphase. We now want to address whether we can manipulate the monolayer extension and thus internal microstructure by adding a linear PNIPAM homopolymer, which is expected to show similar adsorption energy at the air/water interface. Notably, while complete avoidance of contamination is challenging, the chemical identity between linear PNIPAM and the microgel shells ensures that any residual PNIPAM on substrates or in monolayers does not introduce extrinsic impurities. Furthermore, due to the much larger steric hindrance of polymer chains than the much smaller SDS molecules, we expect that the migration of linear PNIPAM chains into the microgel monolayer will be hindered if the PNIPAM chains leave the exterior interface at all. The results shown in Figure S4 in the Supporting Information reveal very stable and constant interfacial tension values for different amounts of linear PNIPAM upon a very short equilibration time of a few minutes. Critically, no spreading agent is required, and desorption from the interface did not occur at these concentrations after a few minutes of equilibration time, eliminating bulk migration concerns.

**Figure 10:**
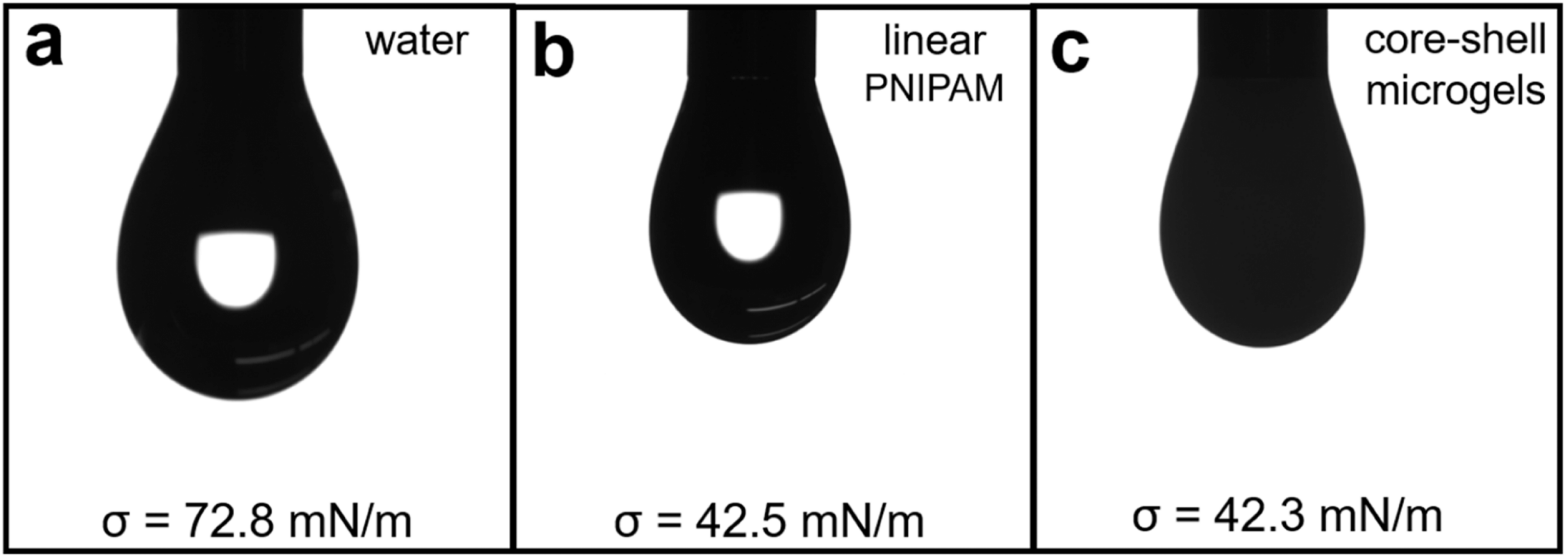
Digital photographs of pendant droplets (aqueous) against air in the steady state. (a) Droplet of pure water. (b) Droplet of a 0.001 wt % aqueous solution of the linear PNIPAM homopolymer. (c) Droplet of a 2 wt % aqueous dispersion of CS_10_ microgels.

We first used pendant drop experiments to compare the steady-state interfacial tension upon self-adsorption at the air/water interface. Figure 10 shows the snapshots of the pendant droplets with pure water (a), an aqueous dispersion of linear PNIPAM homopolymer (0.001 wt %) (b), and the CS_10_ microgels in water (2 wt %) (c). Drop shape analysis revealed an interfacial tension for pure water of 72.8 mN/m in very good agreement with literature values for purified water.^48^ In the case of the dispersions containing either linear PNIPAM or CS microgels, we measured constant values after approximately 10 min, indicating that equilibrium states were reached and that the interface was saturated. In both cases, similar final interfacial tensions of 42.5 mN/m (Figure 10b, linear PNIPAM) and 42.3 mN/m (Figure 10c, CS microgels) were measured. The similarity of these values shows that the reduction of the interfacial tension by PNIPAM is independent of the exact morphology. This points to a similar volume fraction of PNIPAM in the interface volume at equilibrium. Similarly, Zhang and Pelton reported values of approximately 43 mN/m for PNIPAM microgels independent of their cross-linker density.^49^ Thus, it is also safe to assume that the adsorption energy is the same for linear PNIPAM and CS microgels. We now want to study whether the linear PNIPAM homopolymer, when added to the surrounding interface, is suitable for compressing a CS microgel monolayer when normalized to the respective total dimensions. Based on the results from the self-adsorption experiment (Figure 10), we know that the interfacial tension range allowing for manipulation of the monolayer is 42.5–72.8 mN/m. This broad tunability, achieved by adjusting linear PNIPAM amounts (Figure S4), validates its equivalence to SDS in tension modulation within overlapping regimes.

**Figure 11:**
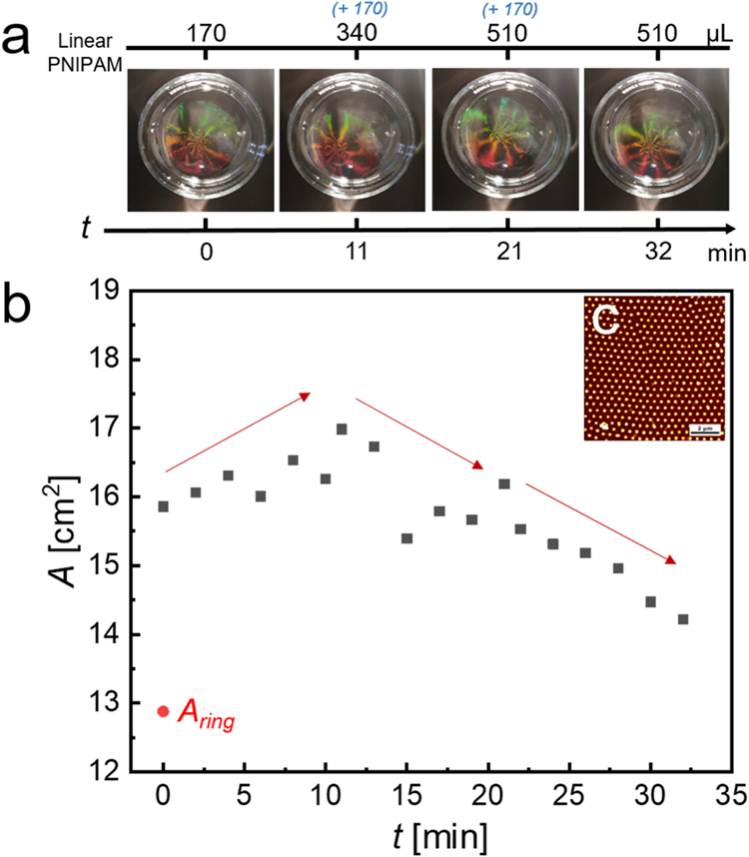
Time-dependent evolution of the CS_10_ microgel monolayer area by adding a linear PNIPAM solution outside the monolayer. (a) Photographs of the monolayer taken at different steps of manipulation through the sequential additions of linear PNIPAM (0.001 wt %, 170 μL per injection) to the surrounding interface. Total volumes of the added PNIPAM solution in microliters are given as black numbers at the top. (b) Time-dependent evolution of the monolayer area, *A*. The red arrows aim to highlight the observed trends in changes in the monolayer area. The red point (*A*_ring_) indicates the area defined by the PTFE ring (calculated by the outer radius of the ring). (c) AFM height image of the monolayer transferred at the final time point (32 min).

Figure 11 shows how we can manipulate a CS microgel monolayer through the subsequent addition of linear PNIPAM to the surrounding interface. The area defined by the PTFE ring (*A*_ring_) was 12.87 mm^2^, which was calculated by the outer radius of the ring. About 170 μL of a solution of linear PNIPAM (0.001 wt %) was added outside the PTFE ring and equilibrated for 10 min. Before removing the ring, the interfacial tension within the monolayer was 49 mN/m, and in the surrounding region containing the linear PNIPAM, it was 61 mN/m. After removing the ring (*t* = 0 min), the monolayer area immediately expanded to 15.85 mm^2^. In the next 10 min, the monolayer area slowly increased to 16.26 mm^2^, driven by the higher interfacial tension outside the monolayer until the interfacial tension imbalance is vanished. Then, at *t* = 11 min, we performed a second addition of linear PNIPAM to the monolayer-free area by injecting another 170 μL of 0.001 wt % solution. This initiated compression of the monolayer from a total area of 16.98 to 16.18 mm^2^ within 10 min. In this case, we have now started with a lower interfacial tension in the surrounding, leading to the compression of the monolayer. Finally, we performed a third addition of linear PNIPAM, this time with another 170 μL injected at *t* = 21 min. This leads to another compression until a final monolayer area of 14.21 mm^2^ is reached over the course of 10 min. The central part of the monolayer was transferred onto a solid substrate at the final state (*t* = 32 min) in order to study its microstructure by microscopy in the dried state (ex situ). The monolayer exhibits hexagonal order in the final state with *d*_c-c_ = 469 nm (Figure 11c), which is very close to the value of the equilibrium starting condition (Figure 4). These experiments confirm that the imbalance in interfacial tension is crucial for the behavior of the monolayer of soft microgels and that the observed effects are rather independent of the architecture and size of the molecules, influencing the interfacial tension outside the microgel monolayer.

## 4 Conclusions

The structure and dynamics of freely floating monolayers of core–shell microgels at air/water interfaces were manipulated by the external interfacial tension, i.e., the interfacial tension outside the monolayer-covered area. In this work, we adjusted the external interfacial tension via the addition of sodium dodecyl sulfate or a linear PNIPAM homopolymer. In the case of significant imbalances in tension between the interface covered with the microgel monolayer with respect to the monolayer-free surrounding, we found either expansion or compression of the monolayer depending on whether the surrounding has a higher or lower interfacial tension. This response is attributed to a pronounced Marangoni flow. When the interfacial tension outside the monolayer is close to the one within, neither expansion nor compression is observed, and center-to-center distances in the monolayer remain nearly constant over time. Furthermore, we found greater monolayer expansion for less cross-linked microgels, consistent with their higher softness determined by dynamic light scattering in bulk. By using the PNIPAM homopolymer to control the external interfacial tension, we could finally demonstrate the stepwise manipulation of the monolayer in a completely surfactant-free system.

This work sheds light on the response of soft colloidal monolayers at fluid interfaces to imbalances in interfacial tension and the role of softness. Our findings provide an alternative strategy for controlling interparticle distances in microgel monolayers at fluid interfaces compared to the typically used Langmuir trough setups where the state of the monolayer is controlled by compression through movable barriers. Being based on readily available standard lab equipment, our approach is notable for its efficiency in terms of both time and cost.

## Data Availability

Data are available from the authors upon reasonable request.

## Supporting Information Available

TEM and SAXS data of silica cores, photos of the 3D printed frame, expansion isotherm, interfacial tension data for the PNIPAM homopolymer, and AFM images (nonequilibrium experiments) (PDF)
