# Supplementary material for: Core–Shell Microgels at Air/Water Interfaces: Role of Interfacial Tension in Monolayer Evolution
Source: Langmuir. 2025 Mar 31;41(14):9274–87. doi: 10.1021/acs.langmuir.4c05050 (PMC12004919; doi:10.1021/acs.langmuir.4c05050)
Supplement: Supplementary file 1 — la4c05050_si_001.pdf [file la4c05050_si_001.pdf]

SUPPORTING INFORMATION

Core-shell microgels at air/water interfaces: Role of interfacial tension on monolayer evolution

*Yichu Zhou,[a] Jérôme J. Crassous,[b] and Matthias Karg[a,c]**

[a]Institut für Physikalische Chemie I: Kolloide und Nanooptik, Heinrich-Heine-Universität Düsseldorf, Universitätsstr. 1, 40225 Düsseldorf, Germany

[b]Institut für Physikalische Chemie, RWTH Aachen University, Landoltweg 2, 52074 Aachen, Germany

[c]Martin Luther University Halle-Wittenberg, Institute of Chemistry, Physical Chemistry of Functional Polymers, 06120 Halle (Saale), Germany

*Email: matthias.karg@chemie.uni-halle.de

Table of Contents

Figure S1. TEM image and SAXS results of silica cores..S2

Figure S2. 3D-printed frame..S3

Figure S3. Expansion isotherm of CS_{10} microgels...S4

Figure S4. Interfacial tension of PNIPAM homopolymer at air/water interface.....................S5

Figure S5. Evolution of monolayer microstructure in non-equilibrium experiments.............S6

TEM image and SAXS results of silica cores

TEM imaging was performed using a JEOL JEM-2100Plus microscope operating in bright-field mode at an acceleration voltage of 200 kV. A dilute aqueous solution of silica particles was drop-cast onto carbon-coated copper grids (200 mesh, Science Services) and allowed to dry at room temperature for at least 1 hour before imaging. **Figure S1a** shows a representative TEM image (bright field) of the spherical silica cores.

SAXS measurements were carried out on a Xeuss 2.0 system (XENOCS) equipped with an X-ray source operating at 8.048 keV (Cu K-Alpha), corresponding to a wavelength of 0.154 nm. The sample-to-detector distance was set to 2.5 m, and the scattering data were collected using a PILATUS3 300 K detector (DECTRIS) with pixel size of 172 × 172 μm^2. A dilute aqueous dispersion of silica particles was loaded into 1 mm round capillaries provided by WJM Glas, and the exposure time was 3600 s. Milli-Q water was used for background correction. The red line in **Figure S1b** is a fit to the measured data (symbols) using the form factor of polydisperse spheres with Gaussian polydispersity. The fit yields an average diameter of 105 ± 6 nm.

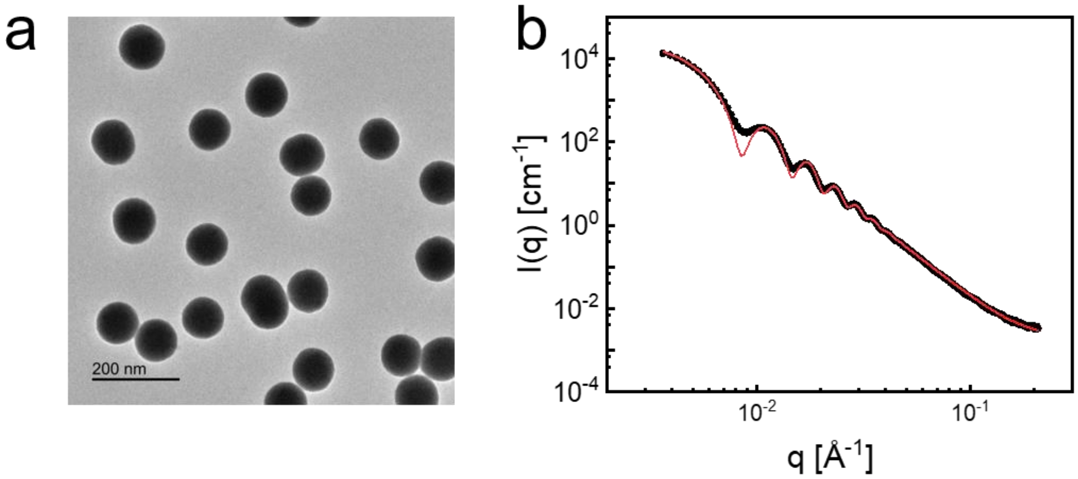

Figure S1. (a) TEM image of silica cores used for the CS microgel synthesis. (b) Measured SAXS profile (black symbols) of the silica NP cores and a corresponding fit to the data (red line).

3D-printed frame

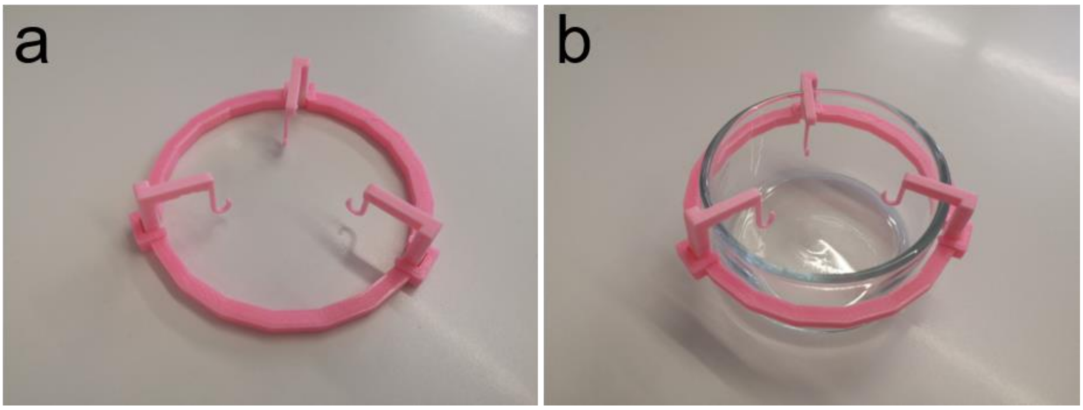

Figure S2. (a) A 3D-printed frame with multiple hooks, designed for suspending the PTFE ring. (b) The frame placed on a crystallizing dish. The frame can be easily lifted by holding the circular part of the frame.

Expansion isotherm of CS_{10} microgels

Figure S3 shows the expansion isotherm of CS_{10} microgels at an air/water interface measured in a Langmuir-Blodgett trough. Before the experiment, the Langmuir trough and barriers were cleaned until the impurities were removed. After filling the trough with Milli-Q water, 60 µL of a dispersion of the CS_{10} microgels was injected into the air/water interface between the two barriers. The area between the two barriers was initiated from 40 cm^2 and then expanded to 280 cm^2. A Wilhelmy plate (5×5 mm^2) was placed in the centre of the trough. A 76 mm long, rectangular glass substrate was vertically placed to the interface. During expansion, the moving speed of the barriers was kept constant at 0.77 mm/min. The interfacial tension increased from 43.0 mN/m to 70.5 mN/m.

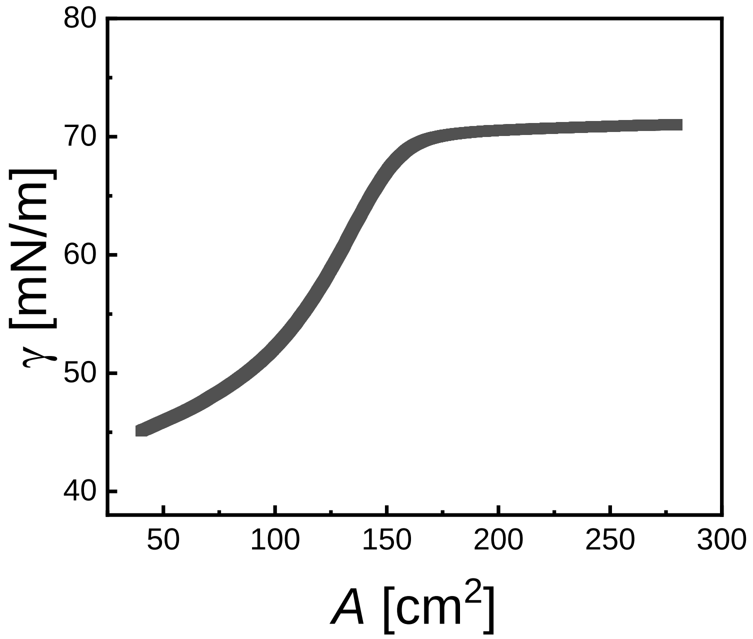

Figure S3. Expansion isotherm of CS_{10} monolayer at the air/water interface in a Langmuir-Blodgett trough.

Time-dependent evolution of interfacial tension for linear PNIPAM homopolymer at the air/water interface

Figure S4 depicts the time-dependent changes in interfacial tension of linear PNIPAM homopolymer at the air/water interface. The spreading solvent consisted of 65 wt% of ethanol and 35 wt% of water. The concentration of the polymer dispersion was 0.01 wt%. First, the crystallizing dish was filled with 85 mL of water. Afterwards, respective amounts of the polymer dispersion were directly injected to the air/water interface. The interfacial tension was measured by a film balance using a Wilhelmy plate (5×5 mm^2). The interfacial tension was measured every 0.2 s upon injection of the polymer dispersion.

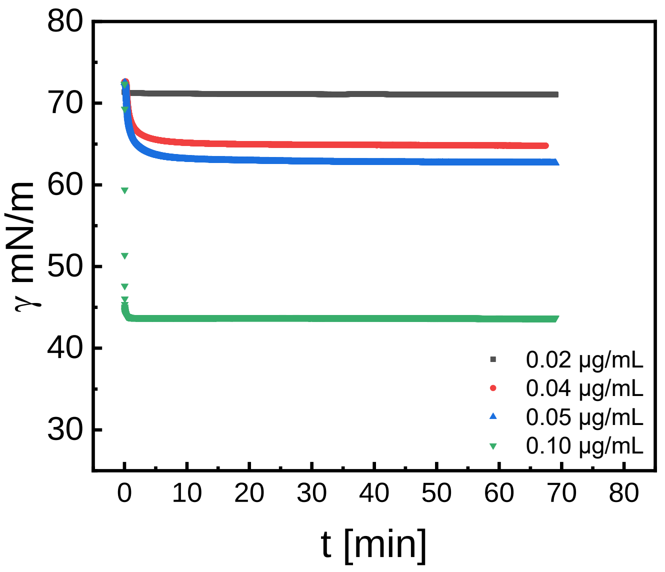

Figure S4. Time-dependent evolution of interfacial tension γ of the air/water interface measured by a tensiometer after adding linear PNIPAM homopolymer. The concentrations of linear PNIPAM are 0.02 µg/mL, 0.04 µg/mL, 0.05 µg/mL, and 0.10 µg/mL, respectively.

Microstructure of the central region of the monolayers over time in non-equilibrium experiments

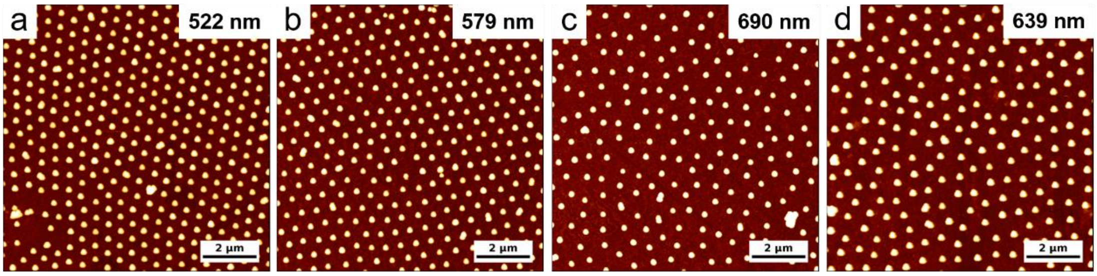

Figure S5. AFM height images of monolayers expanded at the air/water interface after removal of the PTFE ring immediately after SDS addition (0.1 mM). The central parts of the monolayers were transferred to glass substrates at: (a) 30 min, (b) 4 h, (c) 24 h, and (d) 72 h. The determined values of $d_{c\text{-}c}$ are indicated in the top right corner of each panel.
